# Acetone Sensing Properties of a Gas Sensor Composed of Carbon Nanotubes Doped With Iron Oxide Nanopowder

**DOI:** 10.3390/s151128502

**Published:** 2015-11-11

**Authors:** Qiulin Tan, Jiahua Fang, Wenyi Liu, Jijun Xiong, Wendong Zhang

**Affiliations:** 1 Key Laboratory of Instrumentation Science & Dynamic Measurement, Ministry of Education, North University of China, Taiyuan 030051, China; E-Mails: fangjiahua199@163.com (J.F.); wdzhang@nuc.edu.cn (W.Z.); 2 Science and Technology on Electronic Test & Measurement Laboratory, North University of China, Taiyuan 030051, China

**Keywords:** carbon nanotubes, Fe_2_O_3_, gas sensor, acetone

## Abstract

Iron oxide (Fe_2_O_3_) nanopowder was prepared by a precipitation method and then mixed with different proportions of carbon nanotubes. The composite materials were characterized by X-ray powder diffraction, Fourier transform infrared spectroscopy and scanning electron microscopy. A fabricated heater-type gas sensor was compared with a pure Fe_2_O_3_ gas sensor under the influence of acetone. The effects of the amount of doping, the sintering temperature, and the operating temperature on the response of the sensor and the response recovery time were analyzed. Experiments show that doping of carbon nanotubes with iron oxide effectively improves the response of the resulting gas sensors to acetone gas. It also reduces the operating temperature and shortens the response recovery time of the sensor. The response of the sensor in an acetone gas concentration of 80 ppm was enhanced, with good repeatability.

## 1. Introduction

Since the discovery of carbon nanotubes, they have been the focus of nanomaterials research [1]. One-dimensional carbon nanotubes have emerged as important nanomaterials because of their stable chemical properties [2], excellent electrical properties [3,4], larger specific surface area, and strong gas adsorption capacity. These properties have led to their use in making gas sensors that show excellent performance [5,6,7]. However, carbon nanotubes are greatly restricted in their application because of their high cost and nature, which is not conducive to desorption [8,9].

Metal oxide nanopowders are widely used as gas sensing materials [10,11,12,13,14]. Iron oxide is used as a multifunctional semiconductor material, because of its good stability, high sensitivity, low price, and a simple preparation process, which are excellent features for a sensitive material [15,16,17]. But at the same time, it has the disadvantages of a high working temperature, long response recovery time, and low utilization rate. The properties of iron oxide can however be improved by doping it with some other materials like ZnO [18], La [19], Cu [20].

In this paper, the advantages of the two materials, namely carbon nanotubes and iron oxide, are combined to take advantage of their characteristics. Carbon nanotubes can work at room temperature [21], which makes up for the shortcoming of iron oxide of requiring high temperatures to work. Thus, better gas sensitivity of the composite gas-sensitive material is obtained. The purified and chemically modified iron oxide nanopowder-doped carbon nanotube [22], the modification of carbon nanotubes by the iron oxide nanoparticles, and the performance of the gas sensor are investigated through experimentation.

## 2. Experimental Section

### 2.1. Preparation of Gas Sensitive Materials

Carbon nanotubes (350 mg) were heated at 300 °C for 2 h to remove most of the impurities. Further, a mixed solution of concentrated H_2_SO_4_ and concentrated HNO_3_ in a volume ratio of 3:1 was prepared at room temperature. The carbon nanotube powder was added to the mixed acid solution and ultrasonic dispersion was performed for 2 h. The final solution was heated to reflux at 80 °C for 2 h and then cooled to room temperature. Ultrasonic dispersion was then repeated for 3 h. Using a sand core filter and vacuum filtration technique, the carbon nanotubes were repeatedly washed with deionized water until neutral and then dried under an infrared drying lamp for 3 h to obtain chemically modified carbon nanotubes.

An amount of 2.5 g of CO(NH_2_)_2_ was weighed and dissolved in 70 mL of deionized water, while 2.9 g of FeSO_4_·7H_2_O was dissolved in 80 mL of deionized water. The latter solution was stirred and heated to a suitable temperature of 60 °C. The reaction was carried out by dropping the CO(NH_2_)_2_ solution at a speed of 2 mL/min into the FeSO_4_·7H_2_O solution. After completion of the dropwise addition, the reaction was continued for a predetermined time of about 3 min. After stratification, the supernatant was decanted and filtered. The resulting precipitate was washed with deionized water until no SO_2_^−4^ ions were remaining. It was further washed with anhydrous alcohol to remove water and dried to obtain the precursor FeOOH. The obtained precursor was calcined at 200 °C for 2 h, yielding the iron oxide nanopowder sample.

The chemically modified carbon nanotubes were doped with iron oxide nanopowder in mass ratios of 0%, 0.2%, 0.4%, 0.6% and 0.8%, and the doped material was uniformly ground in an agate mortar. Finally, a small amount of deionized water was added dropwise to each sample, to prepare a slurry.

### 2.2. Fabrication of Gas Sensors

**Figure 1:**
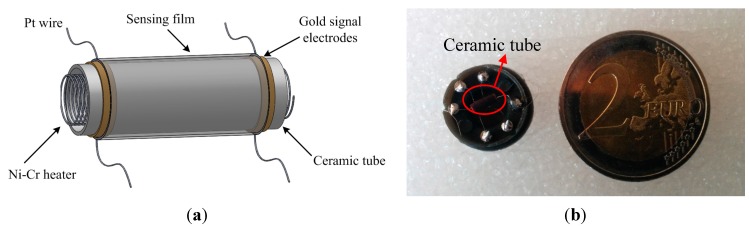
(**a**) Schematic of a gas sensor; (**b**) Illustration of a gas sensor.

The method of preparing a heater-type gas sensor consists of evenly coating the fine slurry on the outer side of the ceramic tube with an Au electrode as shown in Figure 1a. The applied coating should be of even thickness and should be unified at room temperature in a cool dry place, away from direct sunlight to prevent cracking of the gas sensing membrane. After it is completely dry, the ceramic tube is sintered at a temperature of 250 °C for 2 h, which completely dries the gas-sensitive film and strengthens the material, making it more stable. Ceramic tubes with almost same quality was selected for the next experiments. A Pt wire is then welded onto the element base with an Au electrode as shown in Figure 1b. The operating temperature of the sensor is determined by the Ni-Cr heating wire inside the ceramic tube. Finally, the fabricated sensor is aged for 24 h in air, in order to improve its performance.

X-ray powder diffraction (XRD) data was recorded on a Bruker (Karlsruhe, Germany) D8 Advance diffractometer with Cu-Kα radiation (λ = 1.5406 Å), the scanning electron microscopy (SEM) measurements were made on a S-5500 electron microscope (Hitachi, Tokyo, Japan) equipment with an accelerating voltage of 3.0 kV, and the FTIR spectrometer (Spectrum One, PerkinElmer, MA, USA) used KBr tablets. The gas response of the sensor is defined as R_a_/R_g_, where R_a_ and R_g_ are the resistances of the sensor in air and acetone gas, respectively.

## 3. Results and Discussion

### 3.1. Materials Characterization

#### 3.1.1. XRD Analysis

**Figure 2:**
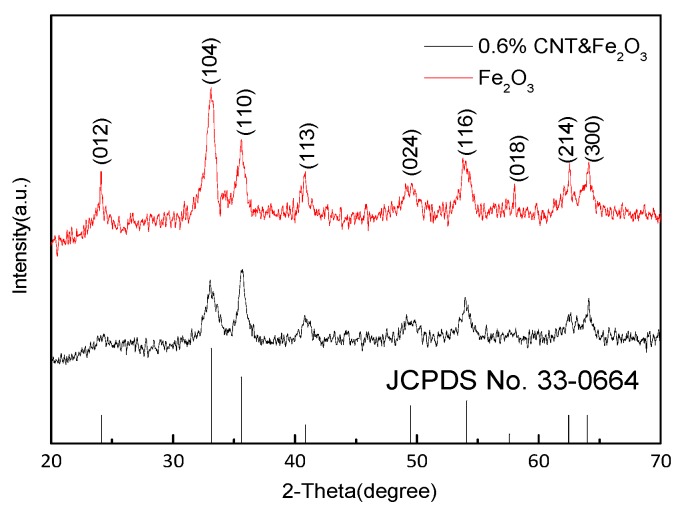
XRD pattern of the iron oxide.

The XRD pattern of iron oxide is shown in Figure 2. Since the iron oxide nanoparticles that were prepared by precipitation have a complete crystal structure, all the diffraction peaks are consistent with the peak of α-Fe_2_O_3_ (JCPDS No. 33-0664), without any impurity peaks. The characteristic diffraction peaks corresponding to the diffraction angles of 24.14°, 33.13°, 35.59°, 40.85°, 49.47°, 54.12°, 57.61°, 62.47°, and 63.97°, further correspond to the crystal planes with indices as (012), (104), (110), (113), (024), (116), (018), (214), and (300), respectively. This indicates that the prepared samples were a pure crystalline form of the rhombohedral structure. It can be seen that the peak of the iron oxide is weakened by the doping with carbon nanotubes, but it is still clear due to the low amount of doping.

#### 3.1.2. IR Analysis

**Figure 3:**
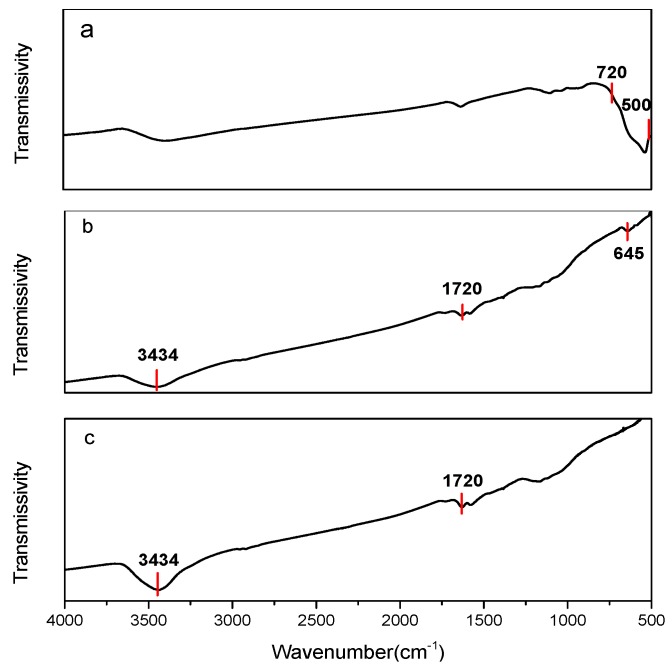
IR spectra of (**a**) nano iron oxide; (**b**) carbon nanotube doped nano iron oxide; (**c**) chemically modified carbon nanotubes.

The carbon nanotubes are boiled in a nitric acid solution. Under heating conditions, the concentrated nitric acid decomposes, releasing nitrogen dioxide and free oxygen which react with the carbon atoms on the carbon nanotubes to form carbon dioxide. This results in refinement and fracture of the carbon nanotubes, generating a corresponding defect and introducing certain functional groups on the carbon nanotubes. Infrared spectroscopy can be used to verify whether the mixed-acid treated carbon nanotubes contain functional groups. Figure 3 shows the IR spectra of the chemically modified carbon nanotubes and iron oxide-doped carbon nanotubes.

Figure 3a–c show the infrared spectra of nano iron oxide and iron oxide nanopowder-doped carbon nanotubes and chemically modified carbon nanotubes, respectively. In both the groups, a strong absorption peak is seen at 3434 cm^−1^, which is the absorption peak of the hydroxyl group (–OH). Another absorption peak is observed at 1720 cm^−1^ which is due to the carboxyl groups (–COOH) introduced through the mixed acid treatment.

In addition, an absorption peak at 645 cm^−1^ also appears in the spectra of carbon nanotube-doped nanometer iron oxide as shown in Figure 3b. Pure Fe_2_O_3_ has a characteristic Fe–O stretching vibration peak at 720–500 cm^−1^ as shown in Figure 3a. When carbon nanotubes and iron oxide are mixed, a new characteristic absorption peak appears at 645 cm^−1^, which corresponds to the characteristic Fe–O stretching vibration peak, proving the existence of Fe_2_O_3._ Also, it shows that the carboxyl groups of carbon nanotubes (–COOH) are covered. The complexity of cooperation of the chemical bond between the iron oxide and carbon nanotubes causes the characteristic Fe–O stretching vibration to shift slightly towards a higher wavenumber. This indicates the successful fabrication of a carbon nanotubes and iron oxide composite.

#### 3.1.3. SEM Analysis

**Figure 4:**
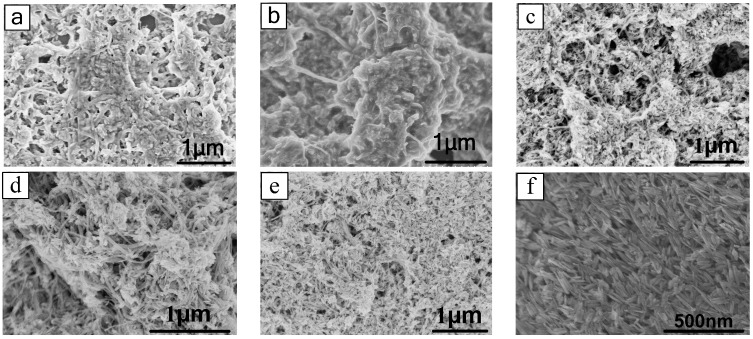
SEM image of (**a**) 0.6% CNT & Fe_2_O_3_ (T = 100 °C); (**b**) 0.6% CNT & Fe_2_O_3_ (T = 150 °C); (**c**) 0.6% CNT & Fe_2_O_3_ (T = 200 °C); (**d**) 0.6% CNT & Fe_2_O_3_ (T = 250 °C); (**e**) 0.6% CNT & Fe_2_O_3_ (T = 300 °C) and (**f**) Fe_2_O_3_ (T = 200 °C).

Figure 4a–e shows the surface morphology of composite gas-sensitive materials which were sintered at different temperatures of 100 °C, 150 °C, 200 °C, 250 °C, and 300 °C, respectively. Figure 4f shows the surface morphology of pure iron oxide. It can be clearly seen in Figure 4a,b that the iron oxide and carbon nanotubes stick to each other if the sintering temperature is too low. Figure 4c,d show a more uniform composite of the two materials that contains a lot of voids that facilitate the absorption of the gas. Also, the sample shown in Figure 4d is better than the sample in Figure 4c. In Figure 4e, most of the particles are iron oxide particles, because the high sintering temperature results in greater loss of carbon nanotubes. Figure 4f shows a sample of pure iron oxide particles that look like needle spindles and are conducive to gas adsorption.

### 3.2. Gas Sensing Performance Tests

**Figure 5:**
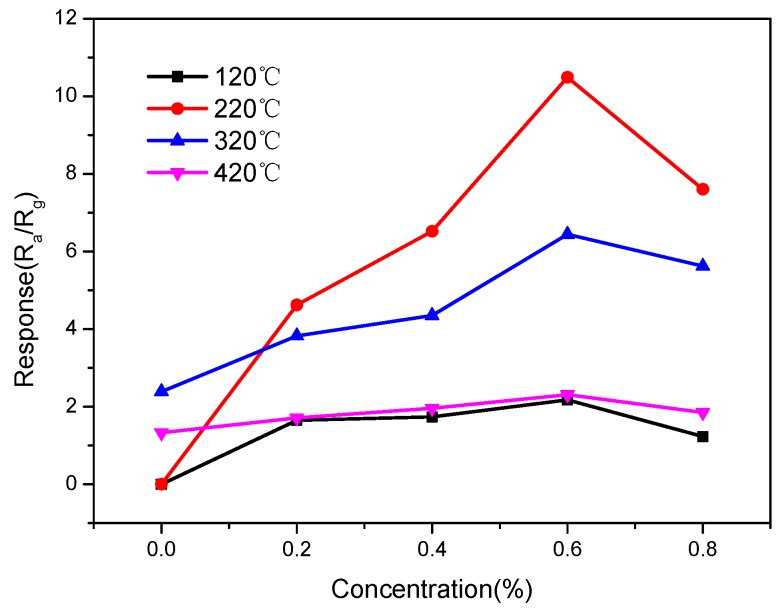
Relationship curves of the response and doping amount.

The response of the gas sensor to an acetone gas concentration of 400 ppm is measured at four different operating temperatures with a doping amount of 0%, 0.2%, 0.4%, 0.6%, and 0.8% of the iron oxide compound that is used as the sensitive material. Figure 5 shows that for a doping amount of 0.6%, the response of sensors at all the four operating temperatures is the highest. When the doping amount exceeds 0.6%, the response of the sensor is reduced by varying degrees for different temperatures. This indicates that the optimum doping amount is 0.6%.

**Figure 6:**
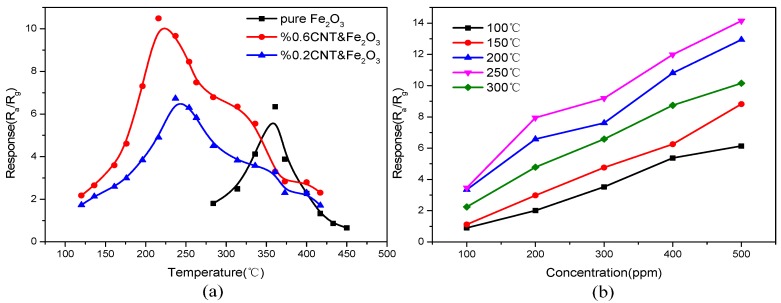
(**a**) Response of different samples in an acetone gas concentration of 400 ppm at various operating temperatures; (**b**) Response of a gas sensor at different sintering temperatures and gas concentrations.

The operating temperature of iron oxide gas sensors is generally higher and the response varies with the temperature. The introduction of carbon nanotubes helps to change the operating temperature of the compound, Figure 6a shows the effect of varying the temperature on the response of the sensor in an acetone gas concentration of 400 ppm for different doping amounts. The response of the sensor increases with increasing temperature till the optimum operating temperature is reached. After this point even as the temperature continues to rise, the response decreases. Comparing the 0%, 0.2%, and 0.6% samples it can be seen that the doped carbon nanotube gas sensors have lower operating temperatures and higher responses as compared to the pure iron oxide gas sensor. It is observed that when the working temperature of the pure iron oxide gas sensor is lower than 283 °C, there is no response and when the working temperature is 361 °C, the maximum response of 6.34 is achieved. However, for a doping amount of 0.6%, the doped gas sensor shows a response at 120 °C, and it reaches the maximum response of about 10.49 at 220 °C.

Iron oxide nanoparticles are relatively small in size and have a high surface activity that provides greater sensitivity. The sintering temperature has a great influence on the sensitivity of the material to gas response as temperature extremes can cause the nanoparticles to grow too large, reducing the surface activity. Therefore, the sintering temperature has a great influence on the gas sensing properties of the material. Figure 6b shows the gas response of a sensor (0.6% CNT & Fe_2_O_3_) in varying gas concentrations at different temperatures. When the sintering temperature is maintained at 250 °C, the response of the gas sensor is higher as compared to other temperatures. Therefore, the optimum sintering temperature is determined to be 250 °C.

**Figure 7:**
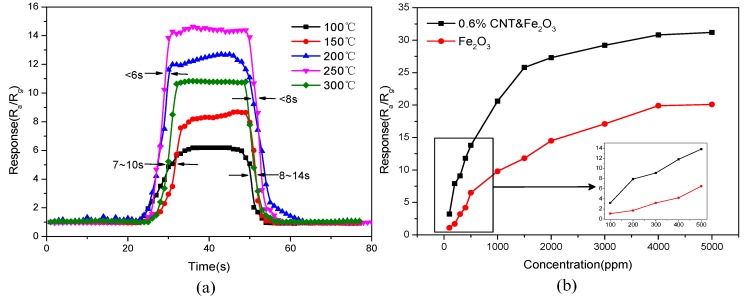
(**a**) Response recovery time at different sintering temperatures; (**b**) Response of sensors in different gas concentrations.

Figure 7a illustrates the sensor response recovery time test curve for different sintering temperatures at an operating temperature of 220 °C in an acetone gas concentration of 500 ppm. It is seen that when the sintering temperature is 250 °C or 200 °C, the sensor response recovery times are less than 6 s and 8 s, respectively. These values are lower as compared to the response recovery times at other sintering temperatures. Thus, it is concluded that 250 °C is the optimum sintering temperature because of the higher response and lower response recovery time of the gas sensor at this temperature.

Figure 7b shows the relationship between the concentration of acetone and the response of the gas sensor with pure iron oxide and the gas sensor with a doping amount of 0.6% at their respective optimum operating temperatures. As can be seen from Figure 7b, the response of the iron oxide-doped carbon nanotube sensors shows a substantial improvement with an increase in the concentration of the acetone gas as compared to the pure iron oxide sensor. These sensors have a more extensive concentration detection range and a good response, especially at low acetone gas concentrations. With further increase in the concentration of acetone, the response shows slower growth. When acetone gas reaches a certain concentration, the sensor gradually reaches a saturation point beyond which the response of the sensor remains considerably unchanged.

**Figure 8:**
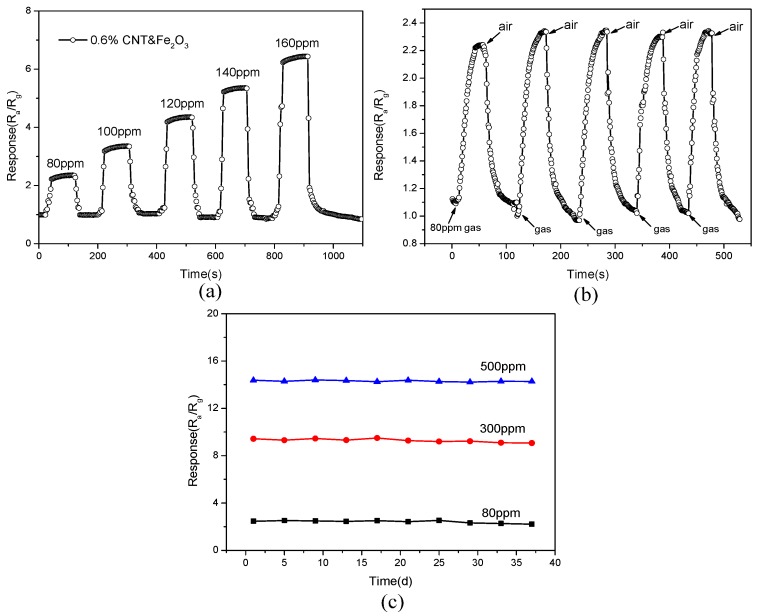
(**a**) Response of the sensor to acetone at low gas concentration; (**b**) The reproducibility of the sensor to 80 ppm acetone at 220 °C; (**c**) Stability of the gas sensor at 220 °C in different concentrations of acetone.

Figure 8a illustrates the curve of the response with the change of time for a sensor (0.6% CNT & Fe_2_O_3_) operating at 220 °C, in the presence of acetone gas with different low concentrations. It is obvious that the response of sensor is about 2.3, 3.2, 4.3, 5.1 and 6.3 to 80, 100, 120, 140, 160 ppm acetone, respectively. In addition the sensor shows a fast response time, when exposed to the acetone and then air. Figure 8b show five reversible cycles of the response recovery time curve. It can be seen that the sensor shows a good response of about 2.34. Further, in the repeated adsorption-desorption experiments, the response recovery time and the response of the sensor remain unchanged, showing good repeatability. Figure 8c shows the stability of the response of a sensor (0.6% CNT & Fe_2_O_3_) with time in different acetone gas concentrations of 80 ppm, 300 ppm, and 500 ppm., It can be seen that during long time continuous operation, response of the sensor remained stable, proving good stability of the gas sensor.

### 3.3. Gas Sensing Mechanism

Iron oxide is an n-type metal oxide semiconductor. At room temperature when this gas sensitive material is exposed to air, a large amount of oxygen molecules come in contact with the sensitive material that capture electrons from the conduction band to form negative oxygen ions [23]. The reaction occurs as shown in Equations (1)–(3). At the same time an electron depletion layer is formed because of the loss of electrons on the surface of the gas sensitive material that results in an increased value of the resistance of the material:

O_2_(gas)→O_2_(ads) (1)

O_2_(ads) + e^−^→O_2_^−^(ads) (2)

O_2_^−^(ads) + e^−^→2O^−^(ads) (3)

When the sensor is placed in acetone gas which is a reducing gas, it reacts with the negative oxygen ions [24,25], as shown in Equation (4). The electron is returned to the surface of the material which results in reduction of the thickness of the electron depletion layer on the surface of the material. As a result, the resistance value of the material also decreases:

CH_3_COCH_3_(gas) + 8O^−^(ads)→3CO_2_(gas) + 3H_2_O(gas) + 8e^−^ (4)

**Figure 9:**
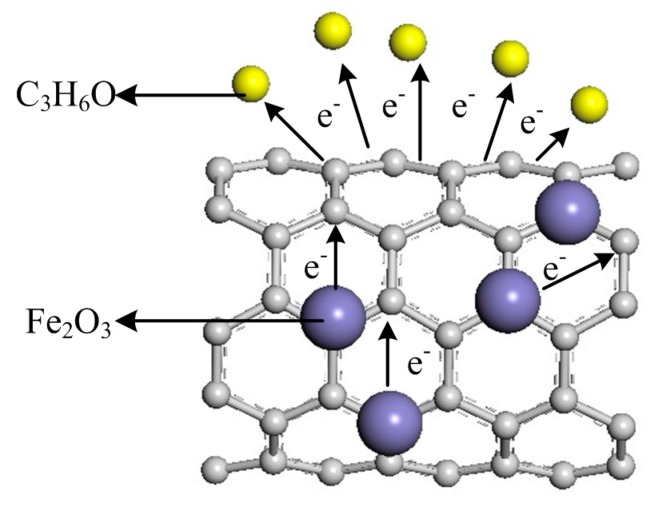
Schematic of the electronic transmission.

Because of the electron work function of carbon nanotubes is greater than that of pure iron oxide, the electrons trapped by oxygen molecules are now provided by iron oxide through carbon nanotubes as shown in Figure 9. The carbon nanotube has a tubular structure and a larger surface area that results in an effective improvement of the adsorption capacity by adsorbing more gas molecules. Thus, the change of resistance value increased, thereby increasing the response of the sensor. On the other hand, the porous structure of carbon nanotubes can effectively speed up the flow of gas, the transfer of electrons, and the change in resistance, thus reducing the response recovery time of the sensors.

Doping of carbon nanotubes which are P-type semiconductors may reduce the resistance of iron oxide which is an n-type semiconductor. When a small amount of carbon nanotubes is added to iron oxide, the increased amplitude of the resistance value is much smaller than the decreased amplitude of the iron oxide resistance value. Thus, the net value of resistance decreases and the response increases. When the added amount of carbon nanotubes exceeds a certain range of quantity, the increase in resistance value of the carbon nanotubes and the decrease in resistance value of iron oxide is almost the same. Thus, even though the resistance change is not obvious, the response is reduced. As a result, there exists an optimum doping amount in the doping process.

## 4. Conclusions

A gas sensor was fabricated by doping carbon nanotubes with iron oxide nanoparticles and then compared with a pure iron oxide gas sensor. The iron oxide-doped carbon nanotube gas sensor shows a significant improvement in the working temperature and response. The response recovery time of the sensor at a doping amount of 0.6% is more rapid, about 4 s and 6.5 s at an optimum sintering temperature of 250 °C and an optimum working temperature of 220 °C. Moreover, the sensor response reaches 2.34 in an acetone gas concentration of 80 ppm. Consequently, the sensor is shown to have a wide detection range and good stability.
